# The ‘new normal’ includes online prenatal exercise: exploring pregnant women’s experiences during the pandemic and the role of virtual group fitness on maternal mental health

**DOI:** 10.1186/s12884-022-04587-1

**Published:** 2022-03-25

**Authors:** Cristina Silva-Jose, Taniya S. Nagpal, Javier Coterón, Ruben Barakat, Michelle F. Mottola

**Affiliations:** 1 AFIPE Research Group, Universidad Politécnica de Madrid, Madrid, Spain; 2 Kinesiology, Faculty of Applied Health Sciences, Brock University St. Catharines, St. Catharines, Canada; 3 School of Kinesiology, Faculty of Health Sciences, University of Western Ontario, London, Canada; 4 Children’s Health Research Institute, University of Western Ontario, London, Canada; 5 Department of Anatomy & Cell Biology, Schulich School of Medicine & Dentistry, University of Western Ontario, London, Canada

**Keywords:** Exercise, Pregnancy, Physical Activity, Coronavirus, Mental health

## Abstract

**Background:**

Prenatal anxiety and depressive symptoms have significantly increased since the onset of the coronavirus (COVID-19) pandemic In addition, home confinement regulations have caused a drastic increase in time spent sedentary. Online group fitness classes may be an effective strategy that can increase maternal physical activity levels and improve mental health outcomes by providing an opportunity for social connectedness. The present study explores the experiences of pregnant women who participated in an online group exercise program during the pandemic and identifies relationships with maternal mental health and well-being. In addition, we present person-informed recommendations on how to improve the delivery of future online prenatal exercise programs.

**Methods:**

Semi-structured interviews were conducted with pregnant women (8-39 weeks of pregnancy) who participated in an online group exercise program, from March to October 2020 in Spain. A phenomenological approach was taken, and open-ended questions were asked to understand women’s experiences throughout the pandemic and the role the online exercise classes may have had on their physical activity levels, mental health, and other health behaviours such as diet. A thematic analysis was performed to evaluate data. In addition, women completed the State-Trait Anxiety Inventory and these data supplemented qualitative findings.

**Results:**

Twenty-four women were interviewed, and the anxiety scores were on average 32.23 ± 9.31, ranging from low to moderate levels. Thematic analysis revealed that women felt safe exercising from home, an increased availability of time to schedule a structured exercise class, and consequently an improvement in their adherence to the program and other behaviours (i.e., healthier diet). Women emphasized feeling connected to other pregnant women when they exercised online together, and overall, this had a positive effect on their mental well-being. Women suggested that future online exercise programs should include flexible options, detailed instructions and facilitation by a qualified exercise professional.

**Conclusion:**

Pregnant women are receptive to online group exercise classes and expressed that they are an accessible option to accommodating physical activity during the pandemic. In addition, the online group environment provides an important sense of connectivity among pregnant women exercising together and this may mitigate the detrimental effect of COVID-19 on maternal mental health.

## Background

Since the declaration of the global coronavirus pandemic (COVID-19) there has been a growing public concern on the impact the virus may have on pregnant women and the developing fetus [1, 2]. Due to the global pandemic, there has been a significant increase in prenatal depressive symptoms, anxiety surrounding labor and delivery, and discomfort attending prenatal hospital visits [3]. Furthermore, given the confinement measures implemented to prevent the spread of COVID-19, pregnant women are experiencing social isolation from family members and other networks that would have normally been a supportive resource during their pregnancy and postpartum [4]. Emerging evidence has shown that prenatal depression has significantly increased since pre-pandemic, and therefore, it is essential to implement effective strategies to support pregnant women and nurture their mental health during this time [5, 6]. One such strategy may be engaging women with physical activity interventions through virtual modalities.

Physical activity during pregnancy improves maternal mental health outcomes, including prevention and treatment of pre- and post-natal depression [7]. Consistently, previous investigations have reported that women who are active during pregnancy have a lower risk of experiencing depressive symptoms compared to women who are mostly sedentary [7–9]. In fact, a large cross-sectional study found that pregnant and postpartum women who were active during the pandemic reported lower levels of depression and anxiety than women who were sedentary [10]. Unfortunately, pregnant women spent over 50% of their day engaged in sedentary behavior prior to the pandemic [11], and it has been suggested that all populations have experienced a significant increase in sedentary time as a result of confinement regulations [12]. In most regions around the world traditional physical activity settings, such as gymnasiums and fitness classes, are inaccessible or have limited capacity as a result of the COVID-19 pandemic [13]. Therefore, alternate modalities need to be explored to offer women opportunities to engage in physical activity while adhering to physical distancing and confinement protocols implemented by public health authorities.

Prenatal group fitness classes are popular during pregnancy as they offer women an opportunity to interact with other pregnant women and increase motivation to be active [14–16]. In accordance with social cognitive and group dynamics theory, group fitness classes have a positive effect on mental health as the setting promotes inclusivity, a shared goal, and a structure that everyone follows together [16, 17]. Various types of prenatal group fitness classes have been examined in a randomized controlled trial design during pregnancy inclusive of aerobic activity, aquatic programs, resistance training, and yoga [18–21]. Irrespective of the type of fitness class, prenatal group fitness appears to improve maternal mental health outcomes and prevent prenatal depression [18–21]. Presently, because of the COVID-19 pandemic virtual delivery of group fitness has become increasingly popular for all population groups [22]. In fact, it was reported in Forbes magazine that COVID-19 may completely re-vamp the fitness industry as there has been a 56% increase in accessing online exercise programs since 2019 [23]. Delivery of prenatal online group fitness may be an accessible and safe way to encourage women to be active during their pregnancy and in the postpartum, and this can protect and improve maternal mental health.

The present study qualitatively explored the experiences of pregnant and postpartum women who participated in online group fitness classes during the pandemic. We aimed to understand and report the perspectives of pregnant and postpartum women who have experienced confinement during pregnancy, and the role online group fitness may have had on their mental and physical health during this time. Additionally, we gathered person-informed suggestions for improving the future delivery of online physical activity interventions for pregnant and postpartum women.

## Methods

This study was established from a phenomenological approach seeking to gain an in-depth understanding of the personal lived experience of the participants [24]. A mixed-method embedded design was used [ 25]. Qualitative procedures and analysis allowed us to establish the main emerging themes narrated by the participants [26]. Quantitative data played a supplemental role to compare and reinforce the results obtained in the qualitative analysis. This study was carried out as a part of a Randomized Controlled Trial (RCT) registered with clinicaltrial.gov (NCT04563065) (24/09/2020) between January 2020 and October 2020. The project was approved by the Ethical Commission of Clinical Research (CEIC) of Hospital Universitario Severo Ochoa and Hospital Universitario Puerta de Hierro (Madrid), and the Ethical Commission of Universidad Politécnica de Madrid (UPM-2020-32/33). The project was performed in accordance with the standards set by the Declaration of Helsinki.

The online supervised exercise program was an adaptation of an in-person moderate physical activity program that was conducted between 8 and 10 and 38-39 weeks of pregnancy [27, 28]. This program was modified and delivered online at the onset of COVID-19 and when the associated physical restrictions were implemented**.** The exercise program was provided by certified fitness professionals 3 times per week for 60 min each session, inclusive of moderate aerobic physical exercise following the structure of the Barakat Model [29]. All classes were delivered in an online format using the Zoom platform.

### Participants

Convenience sampling was carried out within the group of women who attended the online fitness classes during the COVID-19 confinement measures in Spain between March 14 and June 21, 2020 and they continued afterwards until childbirth. The initial sample consisted of 32 women who were attending the online program, of which, 24 consented to be interviewed and are included in the current study. Inclusion criteria were healthy pregnant women over 18 years of age who did not have any type of medical contraindication for exercise, underwent prenatal follow-ups, were receiving normal obstetric care and were attending the online exercise program during this period.

### Procedure

The qualitative design protocol follows the criteria of the COREQ Checklist [30]. Semi-structured interviews were used to learn about the experiences of pregnant women during confinement, the role of participating in online group exercise classes on mental health, and other experiences derived from the health situation generated by COVID-19. Thematic analysis was conducted to examine these data [31, 32].

For the design of the interview, a review of the literature was conducted. This first interview template consisted of 22 questions divided into four blocks (general information about the participants, emotional state, physical activity, and nutrition). In September 2020, 32 women were contacted to request their participation and 26 of them agreed to participate. Two pilot interviews were conducted to check the adequacy of the questions. After the pilot interviews were completed, modifications were made to the interview guide and the final structure with definitive questions was developed. The pilot interviews are not included in the present data analysis, thus the results of this study include 24 participants.

The interviews with the sample of 24 participants were carried out through a computerized platform of video calls (Zoom) between August 31, 2020, and September 24, 2020. The interviews were recorded (audio only) with the permission of the participants. An online form was sent to participants before the interview to collect informed consent, information regarding sociodemographic data and health records.

All women voluntarily participated in the interviews without incentives. All interviews were conducted by the first author and lasted 20-25 min. Each interview was recorded and transcribed textually into a Word document and registered anonymously using a personal identification number. The transcripts were checked for accuracy against the recordings.

Additionally, anxiety was measured using the six-item short-form [33] of the state scale of the Spielberger State-Trait Anxiety Inventory (STAI) [34]. The scale was given to the participants between March and April 2020. STAI is a self-administered questionnaire designed to assess levels of state anxiety by using a Likert-scale (1 to 4). Consequently, higher scores on the scale are associated with higher levels of anxiety. A STAI score of 34-36 is considered normal [33]. STAI scores were included in the present analysis to further describe the population interviewed and supplement the qualitative findings.

### Data analysis

Qualitative data were processed using NVivo Pro 12 (QSR International, Burlington MA) software [35]. Data analysis was carried out following steps proposed by Nowell et al. [36]

The three expert researchers worked together throughout the study to evaluate data, applying a triangulation process to all phases of the analysis. After a generic reading of all the interviews, the basic rules for code generation were established. To assess reliability, part of the interviews were assigned to two researchers at the same time. Subsequently, two meetings were held to agree on the main emerging themes. Each researcher made the assignment of codes to the topics independently and two new meetings were held to review the topics and establish the final structure, defining each of the themes and establishing the relationships. Finally, results were compiled, and a conceptual map was made to depict relationships between the emerged themes. Direct quotes were selected to illustrate interpretations [31].

Version 25.0 of IBM SPSS for Windows (IBM Corporation, Armonk, NY, USA) was used for all quantitative data analyses. Demographic data were presented using descriptive statistics. Preliminary assessments were conducted using the Shapiro-Wilk test to screen for violations of normality. STAI Questionnaire scores were presented by mean, standard deviation (M + SD), minimum and maximum according to tertiles. The lowest tertile is considered low anxiety, the middle tertile moderate, and the highest tertile high anxiety [33]. Data from continuous variables are presented as means and standard deviations, and those of the nominal variables are presented as frequencies and percentages. For descriptive purposes only, we compared STAI scores between women who agreed to participate in the interviews or not, and between women who tested positive for COVID-19 or not using a Student’s t-test. Significance was accepted as *p* < 0.05.

## Results

Overall, 24 participants were interviewed. The women included in the present analyses were 26 to 42 years old (*M* = 35.38, *SD* = 4.43) and were recruited from the aforementioned hospitals in the Region of Madrid. Additional maternal characteristics are shown in Table 1.

**Table 1:** Maternal Characteristics

Characteristics	Frequency (n)	Percentage (%)
**COVID-19 disease**		
No	19	79.16%
Yes	5	20.83%
**Parity**		
None	20	83.3%
One	2	8.3%
Two or more	2	8.3%
**Smoking before pregnancy**		
No	1	4.20%
Yes	23	95.80%
**Smoking during pregnancy**		
No	24	100%
Yes	0	0%
**Occupation**		
Active job	8	33.30%
Sedentary job	12	50%
Homemaker	4	16.70%
**Previous miscarriage**		
None	15	62.50%
One	9	37.50%

**Fig. 1:**
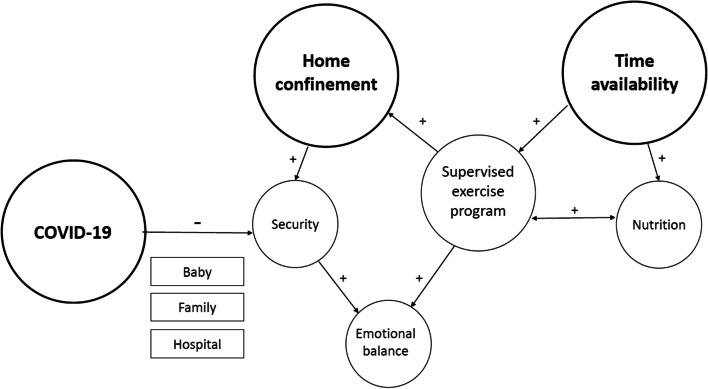
Overview of the main themes and the underlying associations that emerged from the interviews conducted

The findings from the interviews are presented as three themes: time availability, home confinement and COVID-19. Quotes of the study participants are labelled in line with the personal identification number assigned to each interview (Example: PI-1). Figure 1 shows the conceptual map with the relationships of the themes after the analysis of the data. The period of restrictions generated by the pandemic has created a special situation not previously experienced by the population. Three themes emerged clearly connected with the supervised online exercise program. The study participants highlight two realities resulting from the restrictions: on one hand, the availability of more time in their day-to-day life and, on the other, the lack of mobility caused by home confinement. In addition, the experience of the potential threat posed by the presence of the virus and the possibility of infection have nuances that are related and require exploration. Below, we have described each theme and presented representative participant quotes.

### Time availability

The study participants stated that the greater availability of time had beneficial effects on their experience throughout the gestational period, including improvement in their quality of life and accessibility to exercise.

*“It has influenced me a lot; I practice much more exercise than before I was pregnant and confined. When I work, I can’t practice three hours of exercise a week, no way, in fact, my biceps are stronger now than before. So yes, I am grateful that I had more time for doing the physical exercise program. (PI-5).”*

*“In my experience, being confined has helped me to practice more exercise because it has also been an escape route. I was able to keep a weekly rhythm that I would not have had without confinement. I was also able to perform tasks that I could not do in my day-to-day, such as reading or watching movies” (PI-23)*

The increased availability of time is also related to two other aspects that manifested from the interviews: greater adherence to the physically active pregnancy program and an improvement in their eating habits (see below).

#### Adherence to the program

COVID-19 lockdown has been a real advantage in the lives of pregnant women when it comes to engaging in exercise, because participants felt they had more control over organizing their time and could attend all classes within the week. In addition, although the general preference appears to be face-to-face classes, women are receptive to exercising in a group online as well.

*“If I had done it in person, it would have been much better, right? Well, maybe I couldn’t go to Leganés [face-to-face program location], in my case, it would have been complicated. I prefer to go to the place in case you have doubts about the positions.... well, it’s more personal. I prefer face-to-face classes, but during confinement the online program was very good because, otherwise, I would not have done anything, it was a real discovery and it helped me a lot”. (PI-3)*

*“Okay, it did have an influence because, from March until June they already lifted the “punishment” … Well, the truth is that I couldn’t leave the house, exercising personally was an incredible experience for me. It was a hard start to train but once I did, and also my husband did it with me too, it was good. It made me feel good, well, after all, within what we have, we have to adapt to this and, well, practicing exercise and also being able to chat to the moms who talked and told you things it was fantastic. Well, emotional and support levels it has been good “(PI-1)*

Women emphasized that the online exercise classes are a contributing element to achieve optimal physical and mental well-being.

*“Yes, of course, I’m participating in the program and it’s nice and we’re all grateful, but I don’t know how we would be, among the confinement, the extra kilos… In my case, I have stenosis and doctors tell me I shouldn’t gain more than nine kilos during pregnancy, so it’s been fundamental because the program helps me control my weight.” (PI-2)*

Lastly, the study participants mentioned that the virtual group allowed for connectivity, support, and sisterhood, establishing a socio-affective bond with the rest of the participants.

*“I liked very much to see all the bellies moving and even though it is only an hour and we do not know each other but you listen to how others feel: I have gone through this, I have gone through this other, and the experiences that they also told in the group, I enjoyed it very much. (PI-10)*

#### Improved diet

The study participants considered that this time availability has been a necessary resource to be able to eat a healthier and a more complete diet. Confinement has led to changing the type of food they were eating for healthier options, cooking more often and weekly meal planning.

*“I think I cooked, more or less, the same. Maybe I cooked healthier because being here, so long, at home and having more time helps me to cook better and choose healthier and less processed foods. […] and, maybe, I have also cooked more quantity for more days and healthier by having more time." (PI-2)*

*Yes, I changed. I spent more time cooking because I was at home teleworking. We work in a company where they give us food, we have a canteen there to eat, so fortunately, due to COVID we have stopped eating food soaked in oil, so it has been very good for us. I have also not gained excessive weight during my pregnancy, so it has been cool, so… well. ... yes, I’ve changed. (PI-6)*

Finally, a positive connection between attending the supervised online exercise program during pregnancy and an improvement in eating habits was apparent. Women suggested that participating in the class allowed for identification of prior control over their diet, and they felt encouraged to follow dietary guidelines to have a balanced and healthy diet.

*“Well, yes, I think so, because it seems that you stop exercising […], and you enter into a dynamic of… you don't exercise, and you are also eating badly. So, I think yes, it helped, it helped a lot. Besides, it is also a psychological component […] as you are happier, you do everything with more motivation to continue eating well. " (PI-17)*

*"Yes, because you're exercising and, in the end, you encourage yourself to go on a better diet, everything goes together I think, because you start if you let yourself go, you don't exercise or diet or anything". (PI-7)*

### Home confinement

Participants described their pregnancy as ‘peaceful’ and they felt ‘stable’ at home during the confinement period. They felt that home confinement causes a substantial increase in the feeling of freedom from danger while the outside world is perceived as a threat to their pregnancy and postpartum health.

*"Let's see, the truth is that I have not had it ... well, the typical pregnancy. I have not had stress or anxiety or anything, being at home was fine, I would even tell you that it was better for me. " (PI-13)*

*"If I stay two more months locked up at home, nothing happens." (PI-1)*

The study participants state that they have experienced a sense of security and tranquillity within their homes, declaring in most cases that they did not have the need to go outside. In the same way, the exercise program has been used as a strategy to occupy the hours of the day and decrease sedentary behaviours.

*"Yes, the truth is yes. I started the face-to-face with a great enthusiasm. I started the day after they told me. It has served a little to force you to stay active during the confinement. If I hadn't started the program, I would have probably spent the whole confinement lying on the sofa. Maybe climbing the walls, pulling my hair, and saying for goodness's sake, I want to get out of my house! But the truth is... that it has been very good for me. There came a time when you do more sessions because: what do I do? Well, I'm going to do another session, I don't have anything else to do.". (PI-1)*

#### The situation generated by the coronavirus pandemic

COVID-19 generates uncertainty in this population as it is a situation not previously experienced. This fact mainly affects the feeling of safety when different variables come into play that cannot be fully controlled by women, such as the contagion of COVID-19 in their relatives, exposure to their baby and the possible lack of control over their own health during pregnancy, delivery and postpartum. However, virtual exercise classes had an indirect effect on the fear of contracting COVID-19 because women felt healthier because they are physically active. We further elaborate on these findings below.

##### Direct impact of COVID-19 on their relatives and the baby

Within the family group, pregnant women often stressed that the main cause of increased anxiety was caused by worrying about the severity of the virus if a family member were to be infected, fearing deaths among their close relatives. In addition, women who have had children before report feelings of concern and helplessness with the virus potentially infecting their child.

*“I was worried with coronavirus, that my mother, my grandfather, my grandmother had it in my family and I had been in contact with all of them. Then I went into a total panic. " (PI-20)*

*"Yes, in all honesty, my father is currently in the hospital and he has tested positive for COVID. He is asymptomatic, but I am afraid that we might catch it. Furthermore, my father is a dependent person, and he has not left home, so we do not know how he has caught it". (PI-14)*

Relating to the baby, the main concern is of a possible transmission of COVID-19 to their new-born. When the baby is inside the womb, they are perceived as a protective barrier between the baby and COVID-19. However, after labour, an inordinate fear arises when they lose that control over their child.

*"Right now, I am a little more worried because I think I am going to test positive for COVID. I don’t know how it could affect the foetus although everyone has told me that there is no vertical effect, and nothing has to happen. Now, I’m ok. In fact, I have just measured oxygen saturation in my blood, and everything is perfect, so the baby should be perfect, and he moves around a lot, so that is a good sign. (PI-12)*

##### Direct impact of COVID-19 on health emotional balance

The changes that may occur in hospital management due to the evolution of the pandemic have been another source of disruption to women’s perceived safety during the pandemic and women also report feelings of impotence and forgetfulness during this period. Likewise, women report that facing motherhood within these circumstances, generates great confusion and uncertainty about their future, living a new reality where isolation and distance appears to be more encouraged than socialization.

*"That I couldn’t go to the hospital, the need that if something happened, it was not a safe place, paradoxically enough" (PI-18)*

*“In the end, facing motherhood in this situation. All new changes in my life… I feel sorry for the situation. I would love my child to be born, to have a relationship with his family, and the new normality is very cold. I have been suffering from it for a long time and this new reality is terrifying". (PI-21)*

### Suggestions for improving the exercise program

Finally, the study participants understand the limitations of the tools available for exercise during home confinement but add that some better recording and editing tools would increase the quality of the program. Although the general preference is to have face-to-face classes, the women find that the online program is a new reality. To improve future online exercise classes, they emphasized that there should be flexible schedules, thorough explanations of exercises before starting the program, and continuous individualised feedback from qualified trainers.

### Quantitative results

The mean of STAI questionnaire scores was 32.23 ± 9.31 with minimum and maximum values of 17-47. There were no differences in STAI scores compared to women who chose to not participate in the interviews, or when comparing women who tested positive for COVID-19 versus no infection.

The results show that low anxiety was identified in 42.7% and moderate anxiety in 58.3% of the participants. Among the pregnant women interviewed, no high levels of anxiety were recorded (Table 2).

**Table 2:** Descriptive data of scores of women interviewed for each subgroup of the STAI questionnaire

	***n***	M ± SD	Minimum - Maximun
**Low anxiety**	10 / 41.7%	22.33 ± 2.74	17 – 27
**Moderate anxiety**	14 / 58.3%	39.3 ± 4.37	33 - 47
**High anxiety**	0 / 0%	0	0

## Discussion

Pregnant women who experienced confinement and a shift to online prenatal group fitness classes due to the COVID-19 pandemic reported enablers to leading an active lifestyle during this time. Enablers for exercise online include experiencing safety and stability in their home environment and availability of time. Related to this, participants also report an improvement in their dietary patterns by having more time to prepare home cooked nutritious meals. The pandemic situation does indeed increase stress for women, especially due to a lack of certainty on healthcare procedures as they are constantly changing and worrying about the health and well-being of relatives and their newborn. Despite these stressors, overall findings of this investigation suggest that women are successfully able to participate in prenatal group fitness classes online, and there are mental health benefits to being engaged in an online community while in a global pandemic.

Sedentary time has significantly increased over the pandemic as most populations have experienced confinement to their homes and closures of recreational facilities [12]. Pregnant women, who spent over 50% of their day sedentary prior to the pandemic, are a population at risk of excessive sedentary time and low adherence to exercise recommendations [11]. Positively, a large cross-sectional study including 900 pregnant and postpartum women identified that women who maintain an active lifestyle during the pandemic experience lower levels of depressive symptoms [10]. Therefore, it is important to identify outlets for prenatal physical activity during the pandemic and the present study provides evidence that online group fitness is an effective and feasible way to deliver prenatal exercise programs. Moreover, findings from the STAI anxiety scale highlight that this population experienced low to moderate anxiety and this is in line with previous research that has shown active women have better mental health outcomes during the pandemic than women who are sedentary [10]. Findings from our work suggest that pregnant women are receptive to attending online exercise classes and this positively influences their mental health, and in fact, their adherence may be higher than pre-pandemic as time-related barriers have been eliminated.

Lacking time to participate in structured exercise has been a common barrier reported by pregnant and postpartum women, pre-pandemic [15, 37]. Interestingly, in the present analysis women reported more time than before the pandemic to participate in the exercise classes. Given that several studies that have measured activity levels during the pandemic in pregnant and non-pregnant populations report an increase in sedentary time, this finding suggests that perhaps time is not necessarily the barrier during confinement rather other modalities for exercise are needed [38–41]. The present study, included women who were engaged specifically in an online exercise trial with a supervised fitness instructor, and examinations in other populations that have shown reduced activity are from large cross-sectional studies without an exercise intervention.

For example, it was evidenced that sedentary time increased during the pandemic and rates of compliance with the physical activity guidelines during pregnancy [42] were reduced. The number of hours spent sitting increased by 50% during the lockdown, with decreased physical activity as well as self-rated health [43]. Added to this, higher levels of anxiety and depression symptoms were recorded compared to similar pre-pandemic pregnancy cohorts. In these new studies, less physical activity was associated with greater psychological symptoms [44] which, together with home restriction, may be a determinant of depression [45]. This may suggest that exercise interventions delivered in real-time online are key to preventing the collateral consequence of physical inactivity caused by COVID-19.

Furthermore, a recent study that assessed prenatal sedentary time and exercise measured by accelerometers, found that increasing participation in moderate to vigorous exercise does not decrease time spent sedentary throughout the day [46]. This suggests that although adherence to structured online exercise classes may improve due to the availability of time during confinement and virtual options, future studies should build on our work by also targeting the remainder of the day to decrease sedentary time. Irrespective of sedentary time, making time for structured exercise is integral to improving maternal mental health as studies have consistently shown exercising at least 3 times per week at a moderate intensity for a total of 150 min per week can reduce prenatal depressive symptoms and stress [7, 20].

Women reported that causal factors for stress during the pandemic include the constantly changing healthcare environment. Most obstetric and midwifery appointments are delivered by telephone or via online options, especially for low risk pregnancies [47]. Additionally, restrictions have been placed for visiting relatives and this was another cause for stress. The decrease in social support has been identified as a key factor increasing the risk for postpartum depression, maternal anxiety and stress [4, 48]. Positively, our findings allude to online group fitness being an effective modality to still offer women social support in the comfort and safety of their home. Women in late pregnancy also felt feelings of ‘nesting’ as they can securely be at home. Simultaneously, the online classes are offering women an opportunity to be active and interact with other women who are also experiencing pregnancy while in a global pandemic. In line with our findings, several other investigations have reported that prenatal and postpartum group fitness is an effective way to increase socialization, motivation to be active, and leads to positive mental health outcomes [49–51]. Given that women cannot meet in person for exercise, online group fitness can effectively fill this gap and promote both exercise and social support. Of note, despite feeling comfortable at home some women did express wanting to have in person instruction of exercise. This may suggest that future exercise interventions could benefit from a hybrid model incorporating both in person and virtual options to maximize adherence based on the woman’s preference and comfort level.

There is a paucity of research that has examined the effectiveness of in person compared to online delivery of prenatal exercise on health and behavioural outcomes. The few studies that have looked at virtual prenatal exercise options have mostly assessed mobile applications and online education forums [52, 53]. A challenge with these virtual options is poor long-term adherence [54], and this may be due to a lack of real-time engagement as women are expected to use applications or forums in their own time. Our group fitness class was delivered online at a specific time where women were required to log on and attend the class together, with a certified fitness professional leading and supervising the classes. The participants expressed feeling a sense of social support when they logged on to the classes, and overall felt their attendance had actually improved despite the global pandemic. Perhaps real-time delivery of online classes is the best way to mimic in person classes where women would have had to attend at a specific time and would be participating in the class with other women. Moving forward, we may see more online fitness programs for pregnant women and therefore future studies should compare the effect of online versus in-person delivery of real-time group fitness classes on maternal health and behavioural outcomes (e.g., adherence).

Strengths of the present study include the multiple strategies incorporated to establish rigor for qualitative data collection including the purposeful sample of women who were engaged in an exercise intervention during the pandemic, multiple reviewers assessed transcripts to confirm themes, the use of a conceptualization framework, and pilot testing of our interview guide. Limitations include the homogeneity of our sample which does assist with data saturation; however, most women were first time mothers, employed, and all participants were residing in Spain. Consequently, there may be other barriers to online group fitness classes that could not be explored (e.g., parity, socioeconomic status). Findings of this work suggest that pregnant women are receptive to online prenatal exercise classes, however as the popularity of online options increase, perhaps barriers should be identified, and inclusion strategies should be tested to refine programs and encourage all women to be active throughout pregnancy.

## Conclusion

Pregnant women who participated in online group fitness classes during the pandemic reported increased accessibility to being active as they have more time to attend a structured exercise class and are comfortable participating from home. Although they are mildly anxious due to the pandemic, especially because of the constantly changing healthcare system and concerns for their relatives and baby, the online fitness classes offered an opportunity to interact with other women in similar situations and experience social support. Some women do want to return to in-person fitness classes, and therefore, perhaps future delivery of prenatal exercise may include hybrid models of both in-person and online options.

## Data Availability

The datasets generated and/or analysed during the current study are not publicly available due to the maintenance of the signed protocol to ensure the confidentiality of the project’s hospital data but are available from the corresponding author on reasonable request.
